# Probiotics in Pediatrics. A Review and Practical Guide

**DOI:** 10.3390/nu13072176

**Published:** 2021-06-24

**Authors:** Leontien Depoorter, Yvan Vandenplas

**Affiliations:** Vrije Universiteit Brussel (VUB), UZ Brussel, KidZ Health Castle, 1090 Brussels, Belgium; leontien.depoorter@uzbrussel.be

**Keywords:** probiotics, pediatrics, children

## Abstract

The potential benefit of the administration of probiotics in children has been studied in many settings globally. Probiotics products contain viable micro-organisms that confer a health benefit on the host. Beneficial effects of selected probiotic strains for the management or prevention of selected pediatric conditions have been demonstrated. The purpose of this paper is to provide an overview of current available evidence on the efficacy of specific probiotics in selected conditions to guide pediatricians in decision-making on the therapeutic or prophylactic use of probiotic strains in children. Evidence to support the use of certain probiotics in selected pediatric conditions is often available. In addition, the administration of probiotics is associated with a low risk of adverse events and is generally well tolerated. The best documented efficacy of certain probiotics is for treatment of infectious gastroenteritis, and prevention of antibiotic-associated, *Clostridioides* difficile-associated and nosocomial diarrhea. Unfortunately, due to study heterogeneity and in some cases high risk of bias in published studies, a broad consensus is lacking for specific probiotic strains, doses and treatment regimens for some pediatric indications. The current available evidence thus limits the systematic administration of probiotics. The most recent meta-analyses and reviews highlight the need for more well-designed, properly powered, strain-specific and dedicated-dose response studies.

## 1. Introduction

The human microbiome is a topic of great research interest and its role in host protection, physiology and the development of a normal and balanced immune system has been extensively proven. Probiotics are suggested as a therapeutic or preventive option for a variety of childhood diseases [1,2,3].

The term probiotic was defined in 2001 by an Expert Panel of the Food and Agricultural Organization of the United Nations (FAO) and the World Health Organization (WHO). In 2013, the International Scientific Association for Probiotics and Prebiotics (ISAPP) convened an Expert Panel reviewing the field of probiotics and the literature. The result was a consensus statement reiterating the following definition: “Live microorganisms that, when administered in adequate amounts, confer a health benefit on the host”. This is the widely accepted scientific definition of probiotics [4]. 

Increased knowledge and awareness of the possible benefits of probiotics has resulted in an exponential growth of available commercial products in a wide range of forms. Because most of them are classified as food supplements, they have to fulfill less stringent criteria and quality control procedures than medicinal products. This may raise concern about their safety [1,5,6]. Case reports were reported of probiotic administration causing systemic infections and immune stimulation in susceptible populations [7,8]. The possibility of transfer of genes coding for antibiotic resistance has been suggested. Less severe adverse events such as transient gastrointestinal symptoms, including excessive gasiness, bloating or diarrhea, do occur [7,8]. However, adverse events remain exceptionally seldom and are mostly limited to high risk populations. There is overwhelming evidence that probiotics are safe for use in the general population [7,8].

In humans the most frequently used probiotic bacteria belong to the lactic acid-producing genera *Lactobacillus* (including several new genera formerly under the *Lactobacillus* umbrella) or *Bifidobacterium*. Other genera, including *Streptococcus*, *Enterococcus*, *Lactococcus*, *Pediococcus*, *Bacillus* and *Escherichia* and some non-bacterial yeast strains of *Saccharomyces* are also used [9,10].

Due to variation between available studies, including heterogeneity of the strains, the dosage, matrix, administration route, indication, as well as the research protocol, no broad consensus on the use of probiotics could be reached. The American Gastroenterology Association (AGA) and the European Society of Pediatric Gastroenterology, Hepatology and Nutrition (ESPGHAN) recently published different evidence-based recommendations [7,11]. Regarding necrotizing enterocolitis, the AGA, ESPGHAN and the American Academy of Pediatrics (AAP) came to different conclusions [7,12,13]. These caveats result in the necessity for further research. More well designed trials are needed before evidence-based recommendations can be proposed on the indications for pediatric administration of probiotics. 

There is substantial evidence suggesting that the effectiveness of probiotics is specific regarding strain and disease [14]. Moreover, also the matrix in which the probiotic is administered is a factor influencing efficacy [15]. Health-care providers have to consider both factors when prescribing the appropriate probiotic in a given patient. This paper gives an overview on the available literature on the prophylactic and therapeutic use of probiotics in well-defined clinically relevant pediatric conditions to guide pediatricians in decision-making. The focus will be on strain-specific effects of probiotics.

## 2. Methods

We searched the Cochrane Library and MEDLINE for randomized controlled trials (RCT) or their meta-analyses and evidence-based clinical practice guidelines published between 1 January 2000 and 30 April 2021. The search terms used were “infant” and/or “child” and/or “pediatric” and “probiotics” and appropriate search terms for the selected conditions. Only articles in English were selected (Table 1).

## 3. Results: Evidence of Efficacy of Probiotics

### 3.1. Diarrhea

#### 3.1.1. Acute Gastroenteritis

Acute infectious gastroenteritis (AGE) was defined by ESPGHAN as a decrease in the consistency of stools and/or increase in the frequency of evacuations (typically ≥ 3 in 24 h), with or without fever or vomiting [16]. Episodes of acute infectious diarrhea remain a major disease burden in children throughout the world. Most episodes are self-limiting. Treatment includes rehydration with oral rehydration solutions (ORS) and rapid refeeding [16,17]. Meta-analyses and systematic reviews on probiotics in AGE are listed in Table 2. 

A Cochrane review including 63 RCTs in a total of 8014 participants, primarily infants and children, evaluated the effect of administering probiotics for the treatment of AGE in all age groups [17]. The most common organisms evaluated were *Lacticaseibacillus (L.) rhamnosus* GG ATCC53103 (13 studies), *Saccharomyces (S.) boulardii* CNCM I-745 (10 studies) and *Enterococcus lactic acid bacteria* (LAB) SF68 (5 studies). This Cochrane review concluded that “probiotics reduce the duration of diarrhea by approximately 25 h, as well as the risk of diarrhea lasting ≥four days”, but failed to recommend specific strains [17]. The use of probiotics was not associated with any adverse event [17]. However, this review included both pediatric and adult studies and included five studies using dead microbes (which are not defined probiotics, but now called “postbiotics”) [17].

*L. rhamnosus* GG ATCC53103 and *S. boulardii* CNCM I-745 are the two most studied strains. They should be initiated early in the course of acute infectious diarrhea. *L. rhamnosus* GG ATCC53103 should be administered at a minimal dose of 10^10^ colony-forming units (CFU) per day, typically for 5–7 days. The use of *L. rhamnosus* GG ATCC53103 had no effect on stool volume, but is associated with a significantly reduced duration of diarrhea, mean stool frequency on day two and risk of diarrhea lasting ≥four days [2,18]. *S. boulardii* CNCM I-745 should be given at a dose of 250–750 mg a day, for 5–7 days [18]. A recent meta-analysis, including 29 RCTs, concluded that adding *S. boulardii* CNCM I-745 to standard rehydration therapy reduced the duration of diarrhea with one day and efficiency was only seen when administered within 72 h after the onset of symptoms [19]. Another meta-analysis by Feizizadeh et al. showed on the other hand no clear beneficial effect in children with acute diarrhea. Additional studies are required [20]. 

Evidence is also available for *Limosalactobacillus (L). reuteri* DSM 17938, daily dose of 1 × 10^8^ to 4 × 10^8^ CFU for 5–7 days, *L. rhamnosus* 19070-2 and *L. reuteri* DSM 12246, 10^10^ CFU of each strain twice daily for five days [11]. A recent meta-analysis confirmed the reduction of duration of diarrhea and hospitalization when *L. reuteri* DSM 17938 was administered [21]. A RCT in 2002 evaluated the effect of *L. rhamnosus* 19070-2 and *L. reuteri* DSM 12246, 10^10^ CFU of each strain twice daily for five days on acute diarrhea in children in a cohort of 30 children. In the treatment group the duration of diarrhea was significantly reduced [22].

The ESPGHAN Working Group recommended the use of certain probiotics for treating pediatric AGE [11]: *S. boulardii* CNCM I-745 (low to very low certainty of evidence); *L. rhamnosus* GG (very low certainty of evidence); *L. reuteri* DSM 17938 (low to very low certainty of evidence); and *L. rhamnosus* 19070-2 and *L. reuteri* DSM 12246 (very low certainty of evidence). The Working Group recommended strongly against *L. helveticus* R0052 and *L. rhamnosus* R0011 (moderate certainty of evidence) and weakly against *Bacillus clausii* strains O/C, SIN, N/R, and T (very low certainty of evidence) [11], highlighting not only the importance of strain specificity but also of disease specificity.

The AGA on the other hand reported the lack of evidence for the benefit of probiotics in pediatric AGE in a review including 89 studies. Their conclusion is based on the fact that studies performed in other geographical regions cannot be extrapolated to the general population because of differences in genetics, diet, sanitation and endemic enteropathogens [7]. 

A recent Cochrane review included RCT’s comparing a specified probiotic agent with a placebo or no probiotic in patients (adults and children) with acute diarrhea, proven or presumed to be caused by an infectious agent [23]. Eighty-two studies (12,127 participants of whom 11,526 children and 412 adults) were included. This Cochrane review included large trials with low risk of bias and came to a similar conclusion as the AGA [7], that probiotics probably make little or no difference to the number of people who have diarrhea lasting 48 h or longer, and whether probiotics reduce the duration of diarrhea remains uncertain [23]. Last but not least, one more meta-analysis concluded that probiotics (and synbiotics) did not reduce the duration of diarrhea in children in developed countries [24]. Thus, the most recent high-quality reviews all concluded that there is a lack of demonstrated efficacy for probiotics to reduce the duration of diarrhea in children in developed countries.

Regarding prevention of diarrhea, according to a review, *L. reuteri* is reported to be effective in reducing the episodes of AGE in children attending day care centers [25]. The administration of *L. rhamnosus* GG was reported to have the potential to reduce the overall incidence of healthcare-associated diarrhea, such as caused by rotavirus [26]. A RCT assessed the effect of administering *L. reuteri* DSM 17938, 10^8^ CFU daily for three months, to 336 healthy Mexican children. A significant reduction in the number of episodes of diarrhea and days with diarrhea was reported. These effects were seen during the study period and at 3-month follow-up. The number of medical visits, antibiotic use, absenteeism from day school and parental absenteeism were also significantly lower, with important cost savings [27].

In summary: Although older meta-analyses recommend some specific strains to shorten the duration of AGE, the most recent reviews all conclude that there is no evidence of benefits. There is insufficient evidence to recommend the systematic administration of probiotics to prevent AGE.

#### 3.1.2. Antibiotic-Associated Diarrhea

Antibiotic treatment is known to disturb the gastrointestinal microbiome, resulting in a range of symptoms, including diarrhea and crampy abdominal pain. Antibiotic-associated diarrhea (AAD) is defined as three or more liquid stools in 24 h in subjects during or within six to eight weeks after antibiotic treatment and is attributed to the administration of these drugs after exclusion of other possible etiologies. However, not all studies on probiotics in AAD did apply this definition [28]. The risk of AAD is higher in young children and with certain antibiotics, such as aminopenicillins with or without clavulanate, cephalosporines, clindamycin and other antibiotics against anaerobes [28,29]. Meta-analyses and systematic reviews on probiotics in antibiotic-associated diarrhea are listed in Table 3.

A Cochrane systematic review evaluated 33 studies with 6352 children. The participants received probiotics (*Lactobacilli* species, *Bifidobacterium* species, *Streptococcus* species or *S. boulardii* CNCM I-745 or combinations), placebo or other treatments thought to prevent AAD [30]. Global analysis concluded that probiotics may be effective for preventing AAD: the incidence of AAD was 8% in the probiotic group, compared to 19% in the control patients [30]. *L. rhamnosus* GG ATCC53103 and *S. boulardii* CNCM I-745 at 5 to 40 billion CFU per day appear the most appropriate probiotic strains to prevent AAD, but this analysis misses strain-specificity [30]. McFarland and colleagues reported a strain-specific effect in the prevention of adult AAD for *Lactobacillus* species, such as a mixture of *L. acidophilus* CL1285, *L casei* LBC80R, and *L. rhamnosus* CLR2, or *L. casei* DN114001 and *L. reuteri* 55730, while other *Lactobacillus* strains did not show efficacy [14]. Ma et al. evaluated the effect of probiotics on *Clostridium difficile*-associated diarrhea, and concluded based on 11 RCTs, including 4692 patients, that *L. casei* ranked the best in reducing AAD (odds ratio (OR) 0.32, 95% CrI 0.14–0.74) [31]. The impact of probiotic and disease specificity was highlighted in a recent paper in coronavirus disease 2019 (COVID-19) patients [32].

If the use of probiotics for preventing AAD is considered in the presence of risk factors such as the choice of antibiotic agent, duration of treatment, patient’s age, comorbidities, need for hospitalization or previous episodes of AAD, ESPGHAN recommends using *L. rhamnosus* GG ATCC53103 or *S. boulardii* CNCM I-745 [33]. 

In summary: the routine administration of specific strains can be considered in the presence of risk factors such as age of the patient, antibiotic administered, and other comorbidities. However, different guidelines recommend different strains.

#### 3.1.3. *Clostridioides* Difficile-Associated Diarrhea (CDAD)

The majority of AADs are mild to moderate. A fulminant form of pseudomembranous colitis usually develops in children with underlying chronic conditions, such as cystic fibrosis, inflammatory bowel disease or cancer and is due to a causative agent often identified as *Clostridioides* difficile (*C. difficile*) [28,29]. Meta-analyses and systematic reviews on probiotics in CDAD are listed in Table 4.

Moderate-quality evidence suggests that probiotics are associated with a lower risk of *C. difficile* infection [34]. A Cochrane review including 39 RCTs and 9955 participants with 1141 children concluded that administration of probiotics together with antibiotics reduces the risk of *Clostridioides* difficile-associated diarrhea (CDAD) by approximately 60%. In patients at high risk, the beneficial effect of probiotics is even more pronounced [35]. However, this Cochrane review failed to recommend specific strains and does therefore not help to clinical decision making because some strains resulted in a favorable effect while others did not [35].

The AGA does recommend the use of certain strains or combinations in the prevention of *C. difficile* infection, based on the 39 trials evaluated by this Cochrane review, their more recent review in 2020 could not identify any new RCTs. Subgroup analysis of individual probiotics or combinations showed a reduced risk of *C. difficile* infection with *S. boulardii* CNCM I-745; a 2-strain combined product with *L. acidophilus* CL1285 and *L. casei* LBC80R; a 3-strain product with *L. acidophilus*, *L. delbrueckii* subsp. *bulgaricus* and *Bifidobacterium (B.) bifidum* and a 4-strain combination of *L acidophilus*, *L. delbrueckii* subsp. *bulgaricus*, *B. bifidum* and *Streptococcus salivarius* subsp. *thermophilus* [7]. The ESPGHAN Working Group recommends choosing *S. boulardii* CNCM I-745 if probiotics are considered for preventing CDAD [33].

The recent technical review by the AGA, identified five RCTs regarding probiotics as additional treatment to metronidazole or vancomycin in CDAD, showing a benefit for *S. boulardii* CNCM I-745, *L. plantarum* 299v or a 4-strain combined product with *L. acidophilus* ATCC 700396, *L. paracasei* subsp. *paracasei* ATCC 335, *B. animalis* subsp. *lactis* ATCC SD5219 and ATCC SD5219. The fact that *L. rhamnosus* ATCC on the other hand increased the recurrence of *C. difficile* infection highlights again the importance of disease specificity, showing that further studies are needed to identify these probiotic strains as well as patient groups that may benefit [7].

In summary: The routine administration of specific strains can be considered in the prevention and management of CDAD. Although different guidelines recommend different strains, *S. boulardii* CNCM I-745 is recommended in all. 

#### 3.1.4. Nosocomial Diarrhea

Nosocomial or hospital-acquired infections are defined as infections that develop during a hospital stay, meaning that they are not present or incubating on hospital admission. Gastrointestinal infections account for the majority of them [36]. Meta-analyses and systematic reviews on probiotics in nosocomial diarrhea are listed in Table 5.

The ESPGHAN Working Group on Probiotics and Prebiotics provided recommendations on the role of probiotics in the prevention of nosocomial diarrhea in children based on a systematic review of available systematic reviews and RCTs [37]. *L. rhamnosus* GG ATCC53103 administration for the duration of the hospital stay and at a minimal daily dose of 10^9^ CFU reduced the risk of nosocomial diarrhea from 13.9% to 5.2%, including rotavirus gastroenteritis [26]. A RCT, including 90 children, showed that administration of *L. rhamnosus* GG ATCC53103 at 6 × 10^9^ CFU/day in combination with zinc, vitamin B and C during 2 weeks, beginning at the first day of the hospitalization, significantly decreased nosocomial infections (48.9% in the control and 24.4% in the treatment group) [38].

The effect of *L. reuteri* was evaluated in 2 RCTs and these showed no effect on overall incidence of nosocomial diarrhea and symptomatic rotavirus infection. A trial in 2012, including 106 children, found that *L. reuteri* DSM 17938 did not significantly reduce the risk of developing diarrhea or rotavirus infection >72 h after hospitalization [39]. Another research group confirmed in 2015 that administration of *L. reuteri* at a daily dose of 10^8^ CFU for the duration of the hospitalization, was not effective in reducing the incidence of nosocomial diarrhea. Nosocomial diarrhea occurred in 13 of 184 included children, 7 in the treatment group and 6 in the placebo group [40].

A large RCT, including 727 hospitalized children, demonstrated that administration of *B. animalis* subsp. *lactis* failed to prevent nosocomial infections in admitted children who were older than 12 months [41].

In summary: Although there is insufficient evidence to recommend the systematic administration of probiotics to prevent nosocomial diarrhea, all data seem to suggest benefit *for L. rhamnosus* GG ATCC53103.

### 3.2. Functional Gastrointestinal Disorders

#### 3.2.1. Infantile Colic

Infant colic or excessive crying affects 10–30% of healthy infants and their families worldwide. Colic is defined by Wessel’s criteria of crying or fussing for three hours or more a day, for three days or more per week, for three weeks in infants aged less than three months [42]. The natural history is believed to be self-limiting, with symptoms resolving spontaneously by three or four months after birth [43]. Meta-analyses and systematic reviews on probiotics in infantile colic are listed in Table 6.

The majority of available studies have evaluated probiotics as a therapeutic tool. Evidence suggests that in breastfed infants *L. reuteri* DSM 17938 decreases infantile colic, resulting in a mean difference of crying time per day at the age of 3 weeks of 56 min [44,45]. Five RCTs were included in an Cochrane meta-analysis. The majority of the included infants were breastfed and received 1 × 10^8^ CFU of *L. reuteri* DSM 17938 once daily for 21 to 28 days, compared to placebo. Their results showed that probiotic supplementation led to a two-fold greater chance of an at least 50% reduction in daily crying time in colic infants [46]. A recent systematic review, including 20 trials (15 RCTs and five meta-analyses), evaluated the effect of probiotics for the management and prevention of colic. Term infants with an adequate birth weight, without recent antibiotic or probiotic treatment, without evidence of failure to thrive or signs of illness and without major congenital or acquired disorders, were included [47]. The efficacy of *L. reuteri* DSM 17938 was evaluated in 6 RCTs in breastfed infants, showing significantly decreased crying and fussing [47]. It can be concluded that there is evidence that *L. reuteri* given to breastfed infants induced over 50% reduction of duration of crying compared to placebo [44,46,48,49]. A RCT including mainly formula-fed colicky infants treated with daily 1 × 10^8^ CFU *L. reuteri* DSM 17938 could not confirm the beneficial effect [50].

A recent Cochrane review including six RCT’s comparing the prophylactic use of probiotics compared to placebo showed that probiotic supplementation made little or no difference on the occurrence of colic. They do appear to reduce crying time, with the most studied strain, *L. reuteri* DSM 17938, resulting in a reduction of approximately 45 min of daily crying time [43]. Among formula-fed infants, those who received *B. breve* versus placebo had less crying time. Each month of treatment, the difference increased and reached a statistical significance after three months. No significant differences were seen in the breast-fed children [51]. Conversely another research group could not confirm these findings [52]. Researchers also evaluated the daily administration of drops containing *L. reuteri* and vitamin D3 compared to vitamin D3 alone. They reported a significant lower intake of anti-colic medication at the age of three months, associated with less primary care contacts. The infants included were all breastfed, confirmation of these results in formula-fed infants needs to be available before recommending routine use [53].

In summary: there is evidence that *L. reuteri* DSM 17938 effectively reduces infant colic in breastfed infants. No recommendation can be made for formula-fed infants.

#### 3.2.2. Regurgitation

Gastroesophageal reflux (GER) is defined as the passive passage of gastric contents back into the esophagus with or without regurgitation and vomiting, caused by a transient relaxation of the lower esophageal sphincter due to postprandial gastric distension. Gastroesophageal reflux disease (GERD) occurs when GER leads to troublesome symptoms of excessive crying, feeding refusal, failure to thrive, sleep disturbance, chronic cough or opisthotonos [54]. Reflux is extremely common in infants and treatment is based on conservative measures like thickened feedings and upright position after feeding [54,55]. Some probiotic strains are shown to enhance gastric emptying [56]. RCTs on probiotics in regurgitation are listed in Table 7.

*L. reuteri* DSM 17938 prevents regurgitation during the first month of life in breast-fed term infants. A RCT compared 40 infants who received probiotic supplementation or placebo and showed, when treated with *L. reuteri* DSM 17938, 10^8^ CFU daily for four weeks, a decrease in the number of regurgitation episodes a day [57].

Another RCT in 2014 studied the impact of *L. reuteri* DSM 17938 during the first 3 months of life on the onset of colic, reflux and constipation in term children. The study showed a statistical significant difference for regurgitation episodes a day, 2.9 versus 4.6 (*p* < 0.01), in the probiotic and the placebo group, respectively [58].

In a RCT, 42 children with regurgitation were included and divided in a probiotic group and a placebo group. Patients in the probiotic group received 10^8^ CFU of *L. reuteri* DSM 17938 per day for a period of 30 days. Parents noted the frequency of regurgitation at home and gastric emptying time was calculated by an ultrasound at the beginning and the end of the study. The study showed a statistically significant difference in reducing gastric distension, accelerating gastric emptying and thereby diminishing episodes of regurgitation in patients receiving the probiotic strain [59].

In 2017, the safety of a new synbiotic formula, supplemented with *B. lactis* and fructo-oligosaccharides with lactose and a protein ratio of 60% whey and 40% casein, was evaluated. 280 infants were included over a period of 3 months. Results showed a normal growth compared to exclusive breastfed infants and showed a statistical significant decrease of functional constipation (3.2%), regurgitation (10.2%) and infantile crying and colic (10.5%) compared to median prevalence according to the literature (7.8%, 26.7% and 17.7% respectively) [60].

In summary: there is insufficient evidence to recommend a specific strain in the management of regurgitation, although there are some promising data for *L. reuteri* DSM 17938.

#### 3.2.3. Irritable Bowel Syndrome

Irritable bowel syndrome (IBS) is a type of functional abdominal pain disorder (FAPD). IBS is defined by pediatric Rome IV criteria and include all of the following for at least two months prior to diagnosis: abdominal pain for at least four days per month, associated with a change in frequency of stool and a change in appearance of stool [61]. Meta-analyses and systematic reviews on probiotics in IBS are listed in Table 8. In a recent meta-analysis of the epidemiology of pediatric FAPD, including 196,472 children, a prevalence of 13.5% was reported, of which IBS was reported most frequently [62].

The effect of *L. reuteri* DSM 17938 in children with FAPD and IBS has been evaluated in several RCTs with conflicting results [63]. The most recent study compared this probiotic strain to placebo in 54 children with FAPD. It showed that both *L. reuteri* and placebo reduced the frequency and intensity of abdominal pain. However, *L. reuteri*, but not placebo, improved the normal activities of the affected children and their families [64]. Another research group, however, reported the superiority of *L. reuteri* DSM 17938 at 10^8^ CFU used twice daily for four weeks, compared to the placebo [65]. Compared with placebo, *L. rhamnosus* GG ATCC53103 supplementation was associated with a significant higher rate of treatment responders, defined as no pain or a decrease in pain intensity in the overall patient population and in the IBS subgroup [66]. A total of 141 children entered a double blind, placebo controlled RCT and received either *L. rhamnosus* GG ATCC53103 or placebo for eight weeks and entered follow-up for eight weeks. *L. rhamnosus* GG ATCC53103, but not placebo, caused a significant reduction of both frequency and intensity of abdominal pain [67]. *VSL#3*^®^ is a probiotic mixture comprising eight different strains of *Bifidobacterium*, *Lactobacillus* and *Streptococcus* (*L. plantarum*, *L. delbrueckii* subsp. *bulgaricus*, *L. casei*, *L. acidophilus*, *B. breve*, *B. longum*, *B. infantis* and *S. salivaris* subsp. *thermophilus*). VSL#3^®^ is now commercialized as Visbiome^®^ in many countries. A trial compared the effect of six weeks VSL#3^®^ versus placebo in 59 children with IBS. At the end, after a two-week washout period, participants switched to the other group for another six weeks’ treatment. VSL#3^®^ was significantly superior to placebo in the primary endpoint, the subjective assessment of relief of symptoms, as well as in three out of four secondary endpoints: abdominal pain and discomfort, abdominal bloating and gassiness and family assessment of life disruption. No significant difference in stool pattern was seen [68].

In summary: given their safety profile, probiotics seem to be an attractive therapeutic option and clinicians may consider their use, especially of *L. rhamnosus* GG ATCC 53103 and VSL#3^®^ in children with persistent symptoms. However, data are scarce and additional high-quality studies are required before probiotic administration in pediatric IBS can be recommended [69].

#### 3.2.4. Functional Constipation

Functional constipation (FC) is a very common problem in childhood and is defined by the Rome IV criteria [70,71], including two or fewer defecations per week with at least one episode of fecal incontinence per week, a history of retentive posturing or excessive stool retention, a history of painful or hard bowel movements, the presence of a large fecal mass in the rectum and a history of large diameter stools that can obstruct the toilet. Meta-analyses and systematic reviews on probiotics in FC are listed in Table 9.

Probiotics have been shown to significantly increase stool frequency in Asian children in a recent meta-analysis and systematic review. However there was significant heterogeneity between the included trials. Probiotics had no effect on improving stool consistency in children [72]. A recent systematic review on the use of pre-, pro- and synbiotics in the treatment of pediatric FC included 13 RCTs. The majority of the included studies did not demonstrate a significant effect of pre-, pro- and synbiotics on outcome measures such as defecation frequency, fecal incontinence and painful or difficult defecation [73]. Another review also reported no significant difference between the probiotic and the control groups with respect to treatment success. While some studied strains showed some effects on defecation frequency, none of the probiotics had beneficial effects on frequency of incontinence episodes and abdominal pain [74]. Evidence-based recommendations on the treatment of constipation in children developed by ESPGHAN and NASPGHAN (North American Society of Pediatric Gastroenterology, Hepatology and Nutrition) do not support the use of probiotics. This recommendation is based on the evaluation of five RCTs, in which both positive results (*L. rhamnosus* Lcr35, *B. longum* and *L. reuteri* DSM17938) as negative results (*L. rhamnosus GG, B. lactis* strain DN-173 010) were reported. None of the obtained findings were repeated in other trials [75].

In summary: there is insufficient evidence to recommend a specific strain in the management of functional constipation.

### 3.3. Inflammatory Bowel Disease

Inflammatory bowel diseases (IBD) are chronic, relapsing and remitting inflammatory disorders of the gastrointestinal tract. IBD is classified into Crohn’s disease (CD), ulcerative colitis (UC) or IBD-unclassified, the latter meaning that at time of diagnosis it is impossible to categorize the features as either CD or UC [76]. UC is characterized by diffuse inflammation of the colon extending from the rectum proximally. CD can affect any area of the intestinal tract, but most commonly involves the terminal ileum and colon and 20% of the affected children will have perianal involvement, including fissures, fistulas and/or abscesses [77]. 

#### 3.3.1. Ulcerative Colitis (UC)

Meta-analyses and systematic reviews on probiotics in UC are listed in Table 10. In UC, varying results are described when using the probiotic *Escherichia (E.) coli* Nissle 1917 in children. An initial study in 2008 showed promising results, however this was not followed by any RCT in children [78]. In adults, several studies showed a beneficial effect of *E. coli* Nissle 1917 compared to standard treatment with mesalazine alone in maintaining remission of the disease [79]. Again, these results were not confirmed by any RCT [80]. Another frequently studied probiotic in children and adults is VSL#3^®^. In a RCT, VSL#3^®^ has been shown to be effective in inducing and maintaining remission when given as an adjunct to standard therapy with mesalazine; 90% of the children treated with VSL#3^®^ and mesalazine achieved remission compared to 36% in the control group. Moreover, VSL#3^®^ was also considered effective in maintaining remission, because 21% of the patients treated with the probiotic relapsed over the course of one year, whereas 73% of the control group relapsed [81]. Similar results were achieved in an open-label study and VSL#3^®^ was tolerated well by the children without any adverse effects [82]. A small study in children also evaluated the effects of *L. reuteri* ATCC 55730 when administered with oral mesalazine compared to a placebo group. The study showed an increase in remission and an improvement of clinical, endoscopic and histological scores [83]. A recent Cochrane review, including mainly adults in 12 studies, concluded on the other hand that the effectiveness of probiotics for maintenance of remission remains unclear, mainly due to the low quality of the available evidence [84]. An ESPGHAN position paper acknowledged the limited available evidence in favor of these probiotic strains as adjuvant to standard therapy in induction and maintenance of remission [76].

In summary: there is insufficient evidence to recommend the systematic administration of a specific strain in the management of UC, although there are data indicating that *L. reuteri* ATCC 55730 and VSL#3^®^ may be beneficial [85].

#### 3.3.2. Crohn’s Disease

Systematic reviews on probiotics in Crohn’s disease are listed in Table 11. One RCT was performed in 75 children with CD to test the applicability of *L. rhamnosus* GG ATCC53103. Unfortunately, this study showed no beneficial effect of *L. rhamnosus* GG compared to placebo in addition to standard therapy [86] and meta-analysis has shown that *L. rhamnosus* GG can even cause an increase of relapse rate in children [87]. In adults, *S. boulardii* CNCM I-745 was studied and initial studies showed a reduction in relapse rate [88]. However, in a subsequent RCT relapse occurred in 47.5% in the patients treated with *S. boulardii* CNCM I-745, compared to 53.2% in the placebo group. Hence, no statistical significant benefit for *S. boulardii* CNCM I-745 was found in this RCT [89]. The lack of evidence on the benefits of *S. boulardii* CNCM I-745 was confirmed in a recent systemic review [90]. ESPHAN concluded that, as of today, no beneficial evidence is found for the use of probiotics in pediatric Crohn’s disease, hence the workgroup does not recommend the use of probiotics for both the induction or remission of pediatric CD. However, one must consider that currently and especially in children the number of RCTs is limited and further research is required [76].

In summary: there is no evidence that a specific strain may be beneficial in the management of Crohn’s disease.

### 3.4. Helicobacter (H.) pylori

*H. pylori* is a highly prevalent chronic infection causally associated with a spectrum of gastrointestinal disorders, including gastritis, peptic ulcer disease and gastric cancer. The infection is most frequently acquired during childhood. However, in comparison with adults, infected children and adolescents are often asymptomatic and infrequently develop the aforementioned complications. However, spontaneous eradication of *H. pylori* is unlikely and the asymptomatic child may become the symptomatic adult [91]. Meta-analysis and systematic review on probiotics in *H. pylori* are listed in Table 12.

A 2014 meta-analysis, including seven RCTs, evaluated the efficacy of probiotic supplementation in children undergoing *H. pylori* eradication therapy. Compared with the control group, children in the probiotic group experienced a significant increased eradication rate and reduced risk of adverse effects. However there was no standardized protocol of species, dosage and duration of administration in the reviewed studies [92].

*Lactobacilli* as an adjunct to triple eradication therapy have been shown to increase *H. pylori* eradication rates by approximately 13% (71% in the control group versus 84% in the probiotic group). However, eradication rate was still below the recommended goal of 90%. The studied strains differed among reports and included *Lactobacillus acidophilus*, *L. rhamnosus* GG ATCC53103, *L. reuteri* DSM 17938, *L. casei* or compound *Lactobacillus* without further detailed information. Subgroup analysis showed that the eradication rates increased significantly in the high dose group >5 × 10^9^ CFU per day and the long-term group >4 weeks of treatment. *Lactobacilli* supplementation also significantly reduces the risk of diarrhea [93]. 

The efficacy of the probiotic yeast, *S. boulardii* CNCM I-745, was evaluated in a meta-analysis including 11 RCTs with 2200 participants, among them 330 children. In the treatment group, 80% of patients experienced eradication, compared to only 71% in the control group. The addition of *S. boulardii* CNCM I-745 significantly increases eradication rate, but again the eradication rate was still below the goal. The risk of overall side effects was also significantly reduced, particularly of diarrhea and nausea [94].

The fifth edition of the Maastricht consensus on the management of *H. pylori* recommends that certain probiotics may have a beneficial effect on eradication, but the level of evidence was low. Certain strains of the *Lactobacillus* genus and *S. boulardii* CNCM I-745 have shown promising results in reducing gastrointestinal side effects [91].

In summary: there is insufficient data to recommend the systematic administration of a specific strain in the eradication of *H. pylori*, because of the low level of evidence.

### 3.5. Necrotizing Enterocolitis and Late-Onset Sepsis

Necrotizing enterocolitis (NEC) is an ischemic and inflammatory necrosis of the bowel after the initiation of enteral feeding in preterm infants. The incidence of NEC ranges from 2.6% to 28%, with an associated mortality of ~25%. The early clinical presentation includes feeding intolerance, abdominal distention and discoloration and bloody stools [95,96]. The incidence of late-onset sepsis (LOS) is ~20% in very low birth weight (VLBW) infants [97]. Although the pathogenesis of NEC has not been clearly established, intestinal immaturity, insufficient barrier function and dysbiosis with a risk for translocation of pathogens are all involved. In the case of LOS, mechanisms are likely to be similar [98,99]. Meta-analyses and systematic reviews on probiotics in NEC and late onset sepsis are listed in Table 13.

A network meta-analysis for the ESPGHAN Working Group on pre-and probiotics performed a strain-specific review on the available literature [100]. Meta-analyses that group all of the used strains together are already suggesting efficacy; 51 RCTs with over 11,000 preterm infants were included. Most strains or combinations were only reviewed in one or a few RCTs, further large studies with the most promising strains need to be performed to define optimal treatment strategies. Only 3/25 probiotic administrations resulted in reduced incidence of death: *B. bifidum* NCDO 1453 and *L. acidophilus* NCDO 1748 (two studies; 494 preemies); *B. bifidum* and *L. acidophilus* (one study; 186 patients) and *B. infantis*, *L. acidophilus*, *L. casei*, *L. plantarum*, *L. rhamnosus* and *Streptococcus thermophilus* (one study; 150 cases) [100]. According to the analysis, the following seven probiotic combinations resulted in a reduction of the incidence of NEC: *B. lactis* BB12 or B94 (five studies; 828 preemies), *L. reuteri* ATCC 55730 or DSM 17938 (four studies; 1459 patients), *L. rhamnosus* GG ATCC53103 (six studies; 1507 infants), combination of *B. infantis* ATCC 15697 and *L. acidophilus* ATCC 4356 (one study; 367 cases); *B. infantis* BB02, *B. lactis* BB12 and *Streptococcus thermophilus* TH-4 (two studies; 1244 patients) and the combination of *B. longum* 35624 and *L. rhamnosus* GG (two studies; 285 infants) [100]. Two treatments reduced late-onset sepsis, the combination of *B.bifidum*, *B. infantis*, *B. longum* and *L. acidophilus* (two trials with 247 infants) and the combination of *B. longum* R00175, *L. helveticus* R0052, *L. rhamnosus* R0011 and *S. boulardii* CNCM I-1079 (three studies with 241 infants) [100].

The ESPGHAN working group formulated a conditional recommendation (with low certainty of evidence), to administer either *L. rhamnosus* GG ATCC53103 or the combination of *B. infantis* Bb-02, *B. lactis* Bb-12, and *Streptococcus thermophilus* TH-4 in order to reduce NEC rates, if all safety issues are met [12].

The AAP concluded that because of the lack of Food and Drug Administration (FDA)-regulated pharmaceutical-grade products in the United States, conflicting data regarding safety and effectiveness, and possibility to harm in a highly vulnerable population, current evidence cannot support the routine probiotic administration to preterm, especially if birth weight is <1000 g [13].

In a 2020 American systematic review and network meta-analysis of studies to determine the effects of single-strain and multi-strain probiotic preparations on outcomes in preterm, low birth weight infants, superiority of combinations of one or more *Lactobacillus* subsp. and one or more *Bifidobacterium* subsp. was confirmed. The combinations of *Bacillus* subsp. and *Enterococcus* subsp., and one or more *Bifidobacterium* subsp. and *Streptococcus thermophilus* lead to largest reduction of NEC development [101]. In their official recommendation on the use of probiotics in gastrointestinal disorders, AGA suggests using a combination of *Lactobacillus* and *Bifidobacterium* spp., or *B. animalis lactis*, or *L. reuteri,* or *L. rhamnosus* GG ATCC53103 over no and other probiotics in preterm infants [7].

A 2020 Cochrane systematic review included 56 RCT’s in which 10,812 infants participated. Probiotics may reduce the risk of NEC, 33 infants need to be treated to have one additional beneficial outcome. They probably reduce mortality and late-onset invasive infection and have little or no effect on severe neurodevelopmental impairment. Few data were available for extremely preterm (born more than 12 weeks early) or extremely low birth weight infants (less than 1000 g) and analysis did not show any effect on NEC, death or infection. The certainty of this evidence was assessed as being low, due to limitations in trial designs. Further high-quality research is needed, especially for extremely premature and low birth weight infants to provide sufficient evidence of good quality and applicability for practice in this vulnerable population [102].

Concern regarding the safety of probiotics in these fragile patients question the use of probiotics in preterm. As a consequence, daily practice differs substantially in different centers, resulting in a discrepancy in administration from 0 to 100% according to over 150 different neonatal intensive care units [103]. Many trials do not report any adverse event, but some cases of *Lactobacillus* or *Bifidobacterium* sepsis have been reported in infants receiving probiotics [104,105,106,107]. Most affected infants had severe diseases, such as short-bowel syndrome or immunodeficiency [103]. Evidence has shown that preventive measures excluding probiotic administration can result in a decrease in NEC. The risk–benefit ratio depends on the incidence of NEC in a specific neonatal care center since preventive measures excluding probiotics result in a decrease in NEC [108].

In summary: there is insufficient evidence to recommend the systematic administration of a specific strain in the prevention or management of NEC, although there are data reporting beneficial effects.

### 3.6. Allergic Diseases

#### 3.6.1. Atopic Dermatitis

Allergic disease has become a major worldwide health concern. Cow’s milk allergy (CMA) is one of the most common food allergies in early infancy and forms an important part of the atopic march. Atopic dermatitis or eczema, often linked to food allergies such as CMA, is the most common allergic manifestation in infants and young children. Therefore, its occurrence is often the main criterion of efficacy of clinical trials aimed at reducing the allergy burden in infancy. Eczema is an itchy, non-contagious inflamed skin condition [109].

The composition of the gut microbiota has been postulated to play a role in the development, because a balanced microbiome promotes potentially antiallergenic processes: Th1-type immunity, suppression of Th2-induced allergic inflammation and IgA production, which is an essential component of the mucosal immune defense. The increase in allergy prevalence during the last years has been attributed to changes in environmental factors, such as reduced consumption of fermented food, use of antibiotics and other drugs and increased hygiene. The so-called hygiene-hypothesis suggests that a lack of exposure to microbial stimuli in early childhood is a major factor involved in the steep increase of allergy. Vaginal birth or caesarean section, lack of breastfeeding and early use of antibiotics have a significant impact on the colonization patterns of the infant’s gut. Therefore, the manipulation of the microbiota during pregnancy or after birth may have an impact on allergy prevention [110]. Meta-analyses and systematic reviews on probiotics in atopic dermatitis are listed in Table 14.

In 2015, the World Allergy Organization (WAO), published guidelines on prevention of allergic diseases and concluded that there is a possible prophylactic benefit of the use of probiotics in pregnant women at high risk for having an allergic child, or in women who breastfed infants at high risk of developing allergy, or in infants at high risk of developing allergy [3]. Risk factors for developing allergy in a child include a biological parent or sibling with existing or a history of allergic rhinitis, asthma, eczema or food allergy [111]. A systematic review, including 29 trials in which 12 different probiotics or combinations were used, concluded that there are significant benefits of probiotic supplementation in reducing the risk of eczema. Probiotic supplementation does, however, not reduce the risk of other allergic diseases in children [110]. The European Academy of Allergy and Clinical Immunology (EAACI), on the other hand, concluded based on a systematic review of RCTs that there is no sufficient evidence to support the use of probiotics in food allergy prevention [112]. An important limitation of the above mentioned reviews and guidelines is the absence of evidence for probiotic strain, dosage and start and duration of administration. A meta-analysis focusing on *L. rhamnosus* GG ATCC53103 concluded that there was no evidence that administration results in a reduction of the risk to develop atopic eczema [113]. A recent RCT evaluating the efficacy of supplementing mothers from 35 weeks gestation until six months postpartum if breastfeeding and the child until the age of two years found that maternal-only supplementation did not significantly reduce the prevalence of eczema, wheeze or atopic sensitization in the infant by one year. However, they did find a positive impact when the child was given the supplement [114]. *L. rhamnosus* GG was found to be the most researched strain with benefit in a recent systematic review and meta-analysis including 28 studies. The researchers concluded that probiotic supplementation has a positive impact on the prevention on atopic dermatitis [115].

A Cochrane review, which included 39 RCTs, evaluated the effect of probiotic supplementation for treating eczema. The probiotics included, belonged to the Lactobacillus or Bifidobacteria species, alone or in combination, and were administered for four weeks up to six months. The evaluated strains probably make little or no difference in improvement of patient-rated eczema symptoms [116].

In summary: The available evidence suggests that probiotics result in no to limited difference in reduction of atopic dermatitis. However, data suggest a reduction of the severity of eczema. *L. rhamnosus* GG ATCC 53103 is the best studied.

#### 3.6.2. Asthma and Allergic Rhinitis

Asthma is one of the most common chronic respiratory diseases in children and adults. It is characterized by chronic airway inflammation and respiratory symptoms such as wheeze, shortness of breath, chest tightness and cough that vary over time and in intensity, together with variable expiratory airflow limitation [117].

Rhinitis describes inflammation of the nasal mucosa and is clinically defined by symptoms of nasal discharge, itching, sneezing and nasal blockage or congestion. Rhinitis impacts negatively on physical, social and psychological well-being, due to the direct effect of symptoms and the indirect disturbance of sleep with consequent daily fatigue and the use of antihistamines. The commonest form is allergic rhinitis meaning symptoms caused by exposure to an allergen to which the patient is sensitized. Allergic rhinitis can be seasonal or perennial, according to the corresponding allergen [118,119]. Meta-analyses and systematic reviews on probiotics in asthma and allergic rhinitis are listed in Table 15.

A 2013 meta-analysis included 3257 children in nine trials. These trials were heterogeneous in the type and duration of probiotic supplementation and follow-up. The risk ratio of doctor-diagnosed asthma in participating children receiving probiotics was 0.99 and the risk ratio of incident wheeze was 0.97. No evidence to support a protective effect of perinatal use of probiotics in doctor-diagnosed asthma or childhood wheeze was found [120]. A recent meta-analysis, including 19 RCTs with 5717 children, confirmed that probiotic supplementation during pregnancy or early life was not associated with a lower incidence of asthma or wheeze. Subgroup analysis did however show that probiotics significantly reduce wheeze incidence among infants with atopic disease. These results need to be interpreted with caution, due to the small sample size of this subgroup [121]. In a recent animal study, intranasal administration of *L. rhamnosus* GG, but not *Lactobacillus* GR-1, suppressed airway hyperresponsiveness and reduced the counts of eosinophils and Th2-type cytokines in bronchoalveolar fluid [122]. *L. rhamnosus* GG ATCC53103 and *B. lactis* were shown to suppress several aspects of the asthmatic phenotype, such as airway hyperreactivity, antigen-specific IgE production and pulmonary eosinophilia [123].

The results of a long-term study in children were recently published, showing that *L. rhamnosus* HN001 (6 × 10^9^ CFU) was associated with a significant reduction in cumulative wheeze prevalence and a non-significant reduction in cumulative rhinitis prevalence at age 11 years. In a second treatment group, *B. lactis* HN019 (9 × 10^9^ CFU) showed no effect. Probiotics were taken daily from 35 weeks’ gestation to six months postpartum in mothers while breastfeeding and from birth to age two years in infants [124].

Therapeutic effects of probiotics in human asthmatic patients are not well established. A systematic review on the role of probiotics in the treatment of allergic airway diseases includes 12 trials and showed no improvement of quality of life score in asthmatics. Probiotic intake, however, resulted in a longer time free from asthma exacerbations. The included studies on asthma have used only *Lactobacillus* species (*acidophilus*, *rhamnosus* and *casei*) as the probiotic strain with a minimum dose of >10^9^ for at least one month [125]. An eight-week RCT in children with asthma and allergic rhinitis treated with *L. gasseri* A5 as a supplement to standard medications showed a significant reduction in asthma symptoms as well as improvement in objective airway function measurements [126]. Oral administration of *L. rhamnosus* GG alleviated asthma symptoms in an ovalbumine-sensitized model of mouse asthma [127]. Nevertheless, some studies have reported that oral probiotics have little or no clinical effect on allergic diseases, so the current evidence does not support the routine use of probiotics in the treatment of asthma. 

In a 2014 systematic review, five RCTs that addressed the preventive role of probiotics in allergic rhinitis were evaluated. No difference in the incidence of allergic rhinitis between the probiotic and the placebo groups was seen and there was no significant difference in the prevention of allergic rhinitis [128]. Seventeen RCTs including 5264 children were included in a 2019 meta-analysis, which failed to identify a beneficial effect of probiotic supplementation during pre- and postnatal periods on prevention of allergic rhinitis [129].

There have been promising developments in the use of probiotics as an adjuvant treatment in allergic rhinitis [130]. Several systematic reviews and meta-analyses have shown the beneficial effects of probiotics in improving symptoms and quality of life in patients with allergic rhinitis. However, current evidence remains limited due to study heterogeneity [128,131,132]. The administration of *L. acidophilus* 92 in fermented milk significantly improves nasal symptom scores in patients with perennial allergic rhinitis [133]. A placebo-controlled RCT included 60 children with allergic rhinitis of whom half were treated with an antihistamine, together with *L. paracasei*. The other patients received the antihistamine agent with placebo. The treatment group reported an significant improvement in quality of life scores and in nasal itching and sneezing scores [134]. A crossover RCT included 152 subjects between 18 and 45 years of age, with a diagnosis of moderate to severe allergic rhinitis. Subjects received a probiotic supplement Familact^®^ (containing Lactobacilli, *acidophilus*, *casei*, *delbrueckii* subsp. *bulgaricus* and *L. rhamnosus* GG, *B. longum* and *breve* and *Streptococcus salivarius* subsp. *thermophilus*) with intranasal budesonide or intranasal budesonide with placebo for 8 weeks. The addition of probiotics significantly improved quality of life in persistent rhinitis patients [135]. Another research group evaluated the effect of *L. rhamnosus* GG and vitamin D supplementation on the effectiveness of grass-specific sublingual immunotherapy (SLIT) in children. They reported a decrease in symptom-medication score in all groups treated with SLIT, but a significant increase in CD4-, CD25- and Fox3- positive cells in the children receiving SLIT with *L. rhamnosus* GG, corresponding with a better immunologic response [136].

In summary: there is insufficient evidence to recommend probiotic administration to prevent asthma and allergic rhinitis [137].

## 4. Discussion and Conclusions

The gut microbiome plays an important role in health and disease and probiotics represent a promising modality for prophylactic and therapeutic interventions. Several limitations, including the heterogeneity in study designs, small sample size, different strains, various combinations and treatment regimens, limit the evidence of efficacy of probiotics in pediatric diseases [1,7,8]. A general recommendation according to the different indications is listed in Table 16.

Despite these limitations, European guidelines [11,18] currently recommend the use of *L. rhamnosus* GG ATCC53103, *S. boulardii* CNCM I-745, *L. reuteri* DSM 17938, *L. rhamnosus* 19070-2 and *L. reuteri* DSM 12246 in the treatment of AGE. They should be initiated as early as possible in the course of the disease and reduce duration of diarrhea with approximately 24 h. The use of *S. boulardii* CNCM I-745 or *L. rhamnosus* GG could be considered to prevent antibiotic-associated diarrhea [33]. In patients at risk for developing CDAD, the routine administration of probiotics can be considered, the use of *S. boulardii* CNCM I-745 is recommended by several guidelines [7,33].

The administration of *L. reuteri* DSM 17938 has been shown to reduce infant colic in breastfed infants [46,47]. Preliminary evidence suggests possible efficacy of *L. rhamnosus* GG and *VSL#3* in children with persistent symptoms of IBS [67,68,69]. 

There are insufficient data to recommend the systematic use of probiotics in UC. However, some evidence indicates that using VSL#3^®^ or *L. reuteri* ATCC 55730 might be beneficial [81,83]. No sufficient beneficial evidence for the use of probiotic strains in Crohn’s disease has been found [76].

Selected probiotics such as some strains of *lactobacilli* and *S. boulardii* CNCM I-745 may alter the eradication rate and/or risk of gastrointestinal side effects of standard H. pylori treatment, but the level of evidence was assessed as being low [91].

Data reporting beneficial effects of certain probiotic strains in reducing NEC are available and ESPGHAN and the AGA recommends some probiotic strains to reduce NEC [7,12]. ESPGHAN concludes that there was evidence for the following strains or combinations of strains: *L. rhamnosus* GG ATCC53103 or, the combination of *B. infantis* Bb-02, *B. lactis* Bb-12 and *Streptococcus thermophilus* TH-4 [12]. Due to conflicting data, the guidelines of the Committee of the Fetus and Newborn of the APP cannot support the routine administration of probiotics to reduce NEC, particularly in infants with a birthweight <1000 g [13].

There is insufficient evidence for the systematic use of probiotics to prevent or treat asthma and allergic rhinitis [137]. 

In summary: we conclude that benefit of selected probiotic strains for managing or preventing selected pediatric conditions has been demonstrated. The quality of the available evidence, the strain-specificity, and the efficacy depending on influencing factors such as dosage, matrix, duration, route of administration and the indication, currently limit the routine probiotic administration in the pediatric population.

## Figures and Tables

**Table 1:** PICO search strategy.

Population	Infant Child	
**Intervention**	Probiotic therapy	
**Comparison**	Placebo	
	Probiotic therapy	
	Standard treatment	
**Outcome**	AGE	IBD
	AAD and CDAD	*H. pylori*
	Nosocomial diarrhea	NEC
	Infantile colic	LOS
	Regurgitation	Atopic dermatitis
	IBS	Asthma
	Constipation	Allergic rhinitis

Legend: PICO: population, intervention, control, and outcomes; AGE: acute gastro-enteritis; IBD: inflammatory bowel disease; AAD: antibiotic associated diarrhea; CDAD: *Clostridioides* difficile associated diarrhea; NEC: necrotizing enterocolitis; LOS: late onset sepsis; IBS: irritable bowel syndrome.

**Table 2:** Meta-analyses and systematic reviews on probiotics in acute gastroenteritis.

	N° Patients (Children/Adults/NA)	Probiotic	Dose and Duration	Outcome
Allen et al., 2010 Cochrane Review [17]	8014 (6489/352/1173)	*L. rhamnosus* GG ATCC53103 *S. boulardii* CNCM I-745 *Enterococcus* LAB	NA	↓ duration of diarrhea +/− 25 h
Szajewska et al., 2014 ESPGHAN guidelines [18]	NA	*L. rhamnosus* GG ATCC53103 *S. boulardii* CNCM I-745	≥10^10^ CFU/d, 5–7 d 250–750 mg/d, 5–7 d	↓ duration of diarrhea
Szajewska et al., 2020 ESPGHAN guidelines [11]	NA	*L. reuteri* DSM 17938 *L. rhamnosus* 19070-2 and *L. reuteri* DSM 2246	1 × 10^8^–4 × 10^8^ CFU, 5–7 d 10^10^ CFU 2×/d, 5 d	↓ duration of diarrhea
Collinson et al., 2020 Cochrane Review [23]	12,127 (11,526/412/189)	Several	NA	Uncertain effect
Su et al., 2020 AGA guidelines [7]	NA	Several	NA	Recommendation against use of probiotics
Vassilipoulou et al., 2021 [24]	3469 (3469/0/0)	Several	Several	No sufficient clinical impact
In summary: Recent reviews conclude there is insufficient evidence to recommend the systematic administration of probiotics to prevent AGE, although—as listed in the Table above—many meta-analyses and systematic reviews recommend some specific strains.				

Legend: NA: Not available; CFU: Colony-Forming Units; L: *Lactobacillus*; S: *Saccharomyces*; ESPGHAN: European Society of Pediatric Gastroenterology, Hepatology and Nutrition.

**Table 3:** Meta-analyses and systematic reviews on probiotics in antibiotic-associated diarrhea.

	N° Patients (Children/Adults/NA)	Probiotic	Dose and Duration	Outcome
Szajewska et al., 2016 ESPGHAN guidelines [33]	NA	*L. rhamnosus* GG ATCC 53103 *S. boulardii* CNCM I-745	Uncertain >250 mg and <500 mg	↓ incidence of AAD
Guo Q et al., 2019 Cochrane review [30]	6352 (6352/0/0)	*L. rhamnosus* GG ATCC 53103 and *S. boulardii* CNCM I-745	5–40 × 10^9^ CFU/d for the duration of antibiotic treatment	↓ incidence of AAD
Ma et al., 2020 [31]	4692 (NA)	*L. casei*	50–100 × 10^10^ CFU/d	↓ incidence of AAD

Legend: AAD: Antibiotic-associated diarrhea; NA: Not available; CFU: Colony-Forming Units; L: *Lactobacillus*; S: *Saccharomyces*.

**Table 4:** Meta-analyses and systematic reviews on probiotics in *Clostridioides difficile*-associated diarrhea.

	N° Patients (Children/Adults/NA)	Probiotic	Dose and Duration	Outcome
Szajewska et al., 2016 ESPGHAN guidelines [33]	NA	*S. boulardii* CNCM I-745	>250 mg and <500 mg in children	↓ risk of CDAD
Goldenberg et al., 2017 Cochrane Review [35]	9955 (1114/7036/1805)	Several	NA	↓ risk of CDAD
Su et al., 2020 AGA guidelines [7]	NA	Several, but also S. *boulardii* CNCM I-745	NA	↓ risk of CDAD
In summary: There is soms evidence for *S. boulardii* CNCM I-745 to reduce the risk of CDAD.				

Legend: CDAD. *Clostridioides difficile*-associated diarrhea; NA: Not available; CFU: Colony-Forming Units; S: *Saccharomyces.*

**Table 5:** Meta-analyses and systematic reviews on probiotics in nosocomial diarrhea.

	N° Patients (Children/Adults/NS)	Probiotic	Dose and Duration	Outcome
Szajewska et al., 2011 [26]	1092 (1092/0/0)	*L. rhamnosus* GG ATCC 53103	at least 10^9^ CFU/d	↓ risk of nosocomial diarrhea
Hojsak et al., 2018 ESPGHAN guidelines [37]	NA	*L. rhamnosus* GG ATCC 53103	at least 10^9^ CFU/d for the duration of hospital stay	↓ risk of nosocomial diarrhea
In summary: There is evidence for *L. rhamnosus* GG ATCC53103to reduce the risk of nosocomial diarrhea				

Legend: CFU: Colony-Forming Units; L; *Lactobacillus.*

**Table 6:** Meta-analyses and systematic reviews on probiotics in infantile colic.

	N° Patients (Children/Adults/NA)	Probiotic	Dose and Duration	Outcome
Harb et al., 2016 [45]	NA	*L. reuteri* DSM 17938	NA	Effective against colic in breastfed infants
Schreck et al., 2017 [46]	NA	*L. reuteri* DSM 17938	10^8^ CFU/d 21 to 28 days	Effective against colic in breastfed infants
Sung et al., 2018 [44]	345 (345/0/0)	*L. reuteri* DSM 17938	0.2 × 10^8^ CFU/drop, 5 drops orally/d	Effective against colic in breastfed infants
Ong et al., 2019 Cochrane Review [43]	NA	*L. reuteri* DSM 17938	NA	Probably effective against colic in children
Simonson et al., 2020 [47]	NA	Several, but recommending *L. reuteri* DSM 17938	NA	Probably effective against colic in children
In summary: *L. reuteri* DSM 17938 reduces infant colic in breastfed infants.; however, no recommendation can be made in formula fed infants.				

Legend: NA: Not available; CFU: Colony-Forming Units; L: *Lctobacillus.*

**Table 7:** Randomized controlled trials on probiotics in regurgitation.

	N° Patients (Children/Adults/NA)	Probiotic	Dose and Duration	Outcome
Indrio et al., 2011 [59]	42 (42/0/0)	*L. reuteri* DSM 17938	10^8^ CFU/d for 30 d	↓ daily regurgitation
Garofoli et al., 2014 [57]	40 (40/0/0)	*L. reuteri* DSM 17938	10^8^ CFU/d = 5 drops/d for 28 d	↓ daily regurgitation
Indrio et al., 2014 [58]	589 (589/0/0)	*L. reuteri* SM 17938	10^8^ CFU/d = 5 drops/d for 90 d	↓ daily regurgitation (prevention)
Vandenplas et al., 2017 [60]	280 (280/0/0)	*Bifidobqcterium lactis*	10^7^ CFU/g powder	↓ daily regurgitation
In summary: Although there is some data suggesting benefit of *L. reuteri* DSM 17938, the evidence is insufficient to recommend the routine administration of this probiotic in the prevention or managament of regurgitation.				

Legend: NA: Not available; CFU: Colony-Forming Units; L: *Lactobacillus.*

**Table 8:** Meta-analyses and systematic reviews on probiotics in irritable bowel syndrome.

	N° Patients (Children/Adults/NA)	Probiotic	Dose and Duration	Outcome
Guandalini et al., 2010 [68]	59 (59/0/0)	VSL#3^®^	NA, 6 weeks	Improves relief of symptoms
Horvath et al., 2011 [66]	290 (NA)	*L. rhamnosus* GG ATCC 5 3103	1 × 10^9^–3 × 10^9^ 2×/d for at least 4 weeks	Significant higher rate of treatment responders
Pärrty et al., 2018 [63]	NA	*L. reuteri* DSM 17938	NA	Conflicting results
In summary: There are insufficient data to recommend routine administration of probiotic strains in the management of IBS.				

Legend: NA: Not available; CFU: Colony Forming Units. VSL#3^®^, now called Visbiome^®^, is a probiotic mixture comprising eight different strains of *L. plantarum*, *L. delbrueckii* subsp. *bulgaricus*, *L. casei*, *L. acidophilus*, *B. breve*, *B. longum*, *B. infantis* and *S. salivaris* subsp. *thermophilus*); IBS: Irritable bowel syndrome.

**Table 9:** Meta-analyses and systematic reviews on probiotics in functional constipation.

	N° Patients (Children/Adults/NA)	Probiotic	Dose and Duration	Outcome
Tabbers et al., 2014 ESPGHAN & NASPGHAN guidelines [75]	NA	Several	Several	No significant effects
Koppen et al., 2016 [73]	424 (424/0/0)	Several	Several	No significant effects
Huang et al., 2017 [72]	49 (NA)	Several	Several	↑ Stool frequency
Wojtyniak et al., 2017 [74]	515 (515/0/0)	Several	Several	No significant effects
In summary: No meta-analysis or guideline recommends the administration of a specific strain in the management of functional constipation.				

Legend: NA: Not available.

**Table 10:** Meta-analyses and systematic reviews on probiotics in ulcerative colitis.

	N° Patients (Children/Adults/NA)	Probiotic	Dose and Duration	Outcome
Miele et al., 2018 [76] ESPGHAN position paper	NA	*L. reuteri* ATCC 55730 *VSL#3^®^*	NA	Limited evidence of benefit
Iheozor-Ejiofor et al., 2020 [84] Cochrane Review	1473 (NA, mainly adults)	Several	NA	Uncertain benefit
In summary: There is insufficient evidence to recommend probiotics in ulcerative colitis.				

Legend: NA: Not available; CFU: Colony-Forming Units, VSL#3^®^, now called Visbiome^®^, is a probiotic mixture comprising eight different strains of *L. plantarum*, *L. delbrueckii* subsp. *bulgaricus*, *L. casei*, *L. acidophilus, B. breve*, *B. longum*, *B. infantis* and *S. salivaris* subsp. *thermophilus*.

**Table 11:** Systematic reviews on probiotics in Crohn’s disease.

	N° Patients (Children/Adults/NA)	Probiotic	Dose and Duration	Outcome
Sivanthan et al., 2018 [90]	NA	*S. boulardii*	NA	No significant effects
Miele et al., 2018 [76] ESPGHAN position paper	NA	Several	NA	No recommendation
In summary: There is insufficient evidence to recommend probiotics in Crohns’ disease.				

Legend: NA: Not available.

**Table 12:** Meta-analysis and systematic review on probiotics in *H. pylori*.

	N° Patients (Children/Adults/NA)	Probiotic	Dose and Duration	Outcome
Li et al., 2013 [92]	508 (508/0/0)	Several	Several	↑ eradication rate ↓ side effects
Malfertheiner et al., 2016 Maastricht V consensus report [91]	NA	Several *Lactobacillus* strains *S. boulardii*	NA	↓ side effects
In summary: There is insufficient evidence to recommend probiotics in *H. pylori.*				

Legend: NA: Not available.

**Table 13:** Meta-analyses and systematic reviews on probiotics in necrotizing entero-colitis and late onset sepsis.

	N° Patients (Children/Adults/NA)	Probiotic	Dose and Duration	Outcome
van den Akker et al., 2018 [100]	11,231 (11,231/0/0)	Several (see text)	Several	↓ incidence and mortality
Morgan et al., 2020 [101]	15,712 (15,712/0/0)	Several (see text)	Several	↓ NEC development
Sharif et al., 2020 Cochrane Review [102]	10,812 (10,812/0/0)	Several (see text)	Several	↓ NEC, mortality and late-onset invasive infections
In summary: Systematic administration of probiotic bacteria to prevent NEC is still debated in literature; therefore routine administration cannot be recommended.				

Legend: NA: Not available.

**Table 14:** Meta-analyses and systematic reviews on probiotics in atopic dermatitis.

	N° Patients (Children/Adults/NA)	Probiotic	Dose and Duration	Outcome
Muraro et al., 2014 EAACI guidelines [112]	NA	NA	NA	No efficacy in prevention
Cuello-Garcia et al., 2015 [110]	3447 (3447/0/0)	Several (see text)	Several	↓ risk of eczema
Makrgeorgou et al., 2018 Cochrane Review [116]	2599 (NA)	Several (see text)	Several	Little or no difference in eczema symptoms
Szajewska et al., 2018 [113]	889 (NA)	*L. rhamnosus* GG ATCC 53103	Several	No efficacy in prevention
Li et al., 2019 [115]	3595 (3595/0/0)	Several	Several	Prevention of atopic dermatitis
In summary: Insufficient evidence to recommend routine use of probiotics in the treatment of atopic dermatitis; *L. rhamnonsus* GG ATCC 53103 can be considered.				

Legend: EAACI: European Academy of Allergy and Clinical Immunology; NA: Not available.

**Table 15:** Meta-analyses and systematic reviews on probiotics in asthma and allergic rhinitis.

	N° Patients (Children/Adults/NA)	Probiotic	Dose and Duration	Outcome
Ranjan et al., 2010 [131]	610 (357/253/0)	Several (see text)	Several	↑ Quality of life ↓ Episodes of rhinitis/year
Azad et al., 2013 [120]	3257 (3257/0/0)	Several (see text)	Several	No protection against asthma or childhood wheeze
Das et al., 2013 [125]	899 (571/292/36)	Several (see text)	Several	↑ time between episodes of rhinitis and asthma. No improvement in quality of life.
Peng et al., 2015 [128]	NA	Several (see text)	Several	No prevention of allergic rhinits
Du et al., 2019 [129]	5264 (5264/0/0)	Several (see text), *L. rhamnosus* GG	Several	Prevention of asthma
Wei et al., 2020 [121]	5717 (5717/0/0)	Several (see text)	Several	No protection against asthma or childhood wheeze
In summary: No evidence to recommend probiotics to prevent asthma and allergic rhinitis; none of the reviews recommends specific strains.				

Legend: NA: Not available.

**Table 16:** Practical guide for pediatric use of probiotics.

Conditions	Strains	Dose	Recommended
**Acute gastro-enteritis** **Treatment**	*S. boulardii* CNCM I-745	250–750 mg/day, for 5–7 days	?
	*L. rhamnosus* GG ATCC53103	minimal dose of 10^10^ CFU/ day, for 5–7 days	?
	*L. reuteri* DSM 17938	1–4 × 10^8^ CFU/day, for 5–7 days	?
	*L. rhamnosus* 19070-2 *and L. reuteri* DSM 12246	10^10^ CFU of each strain twice daily, for 5 days	?
**Acute gastro-enteritis** **Prevention**	*L. reuteri* DSM17938	10^8^ CFU/day	?
**Antibiotic-associated diarrhea** **Prevention**	*L. rhamnosus* GG ATCC53103	5–40 × 10^9^ CFU/day, for the duration of antibiotic treatment	+
	*S. boulardii* CNCM I-745	>250 mg and <500 mg	+
	*L. casei*	50–100 × 10^10^ CFU/day	+/−
**C. difficile associated diarrhea** **Prevention**	*S. boulardii* CNCM I-745	>250 mg and <500 mg in children	+
**Nosocomial diarrhea** **Prevention**	*L. rhamnosus* GG ATCC53103	>10^9^ CFU/day, for the duration of hospital stay	+
**Infantile colic** **Prevention and Treatment**	*L. reuteri* DSM 17938	10^8^ CFU/day, for 21–28 days	+ in breastfed No in formula fed
**Regurgitation** **Prevention and Treatment**	*L. reuteri* DSM 17938	10^8^ CFU/day, for at least 30 days	No
**Irritable Bowel Syndrome** **Treatment**	*L. rhamnosus* GG ATCC53103	1–3 × 10^9^ 2×/day, for at least 4 weeks	?
	*VSL#3^®^*	NA, at least 6 weeks	No
**Constipation** **Treatment**	No significant effect of probiotics.		No
**Ulcerative Colitis** **Treatment**	*L. reuteri* ATCC 55730	NA	No
	VSL#3^®^	NA	No
**Crohn’s Disease** **Treatment**	No significant effect of probiotics.		No
**H. pylori** **Treatment**	*Lactobacilli* (*acidophilus*, *rhamnosus* GG ATCC53103, *reuteri* DSM 17938, *L. casei*)	NA	No
	*S. boulardii CNCM I-745*	NA	No
**NEC and Late-onset sepsis** **Treatment**	*L. rhamnosus GG* ATCC53103	NA	?
	The combination of *B. infantis* Bb-02, *B. lactis* Bb-12 and *Streptococcus thermophilus* TH-4	NA	?
**Atopic Dermatitis** **Prevention and Treatment**	*L. rhamnosus* GG ATCC53103	NA	No
**Asthma and Allergic rhinitis** **Prevention and Treatment**	No significant effect of probiotics		No

Legend: NA: Not available. VSL#3^®^, now called Visbiome^®^, is a probiotic mixture comprising eight different strains of *L. plantarum, L. delbrueckii* subsp. bulgaricus, *L. casei, L. acidophilus, B. breve, B. longum, B. infantis* and *S. salivaris* subsp. thermophiles; Recommended: Routine administration can be recommended (yes/no/?(= debated in literature).
